# Lockdowns and the COVID-19 pandemic: What is the endgame?

**DOI:** 10.1177/1403494820961293

**Published:** 2021-02

**Authors:** Theodore Lytras, Sotirios Tsiodras

**Affiliations:** 1 National Public Health Organisation, Greece; 2 School of Medicine, European University Cyprus, Cyprus; 3 4th Department of Internal Medicine, Attikon University Hospital, Medical School, National and Kapodistrian University of Athens, Greece

**Keywords:** COVID-19, pandemic, surveillance, social distancing, herd immunity, epidemiology

## Abstract

An overall long-term strategy for managing the coronavirus disease 2019 (COVID-19) pandemic is presented. This strategy will need to be maintained until herd immunity is achieved, hopefully through vaccination rather than natural infection. We suggest that a pure test-trace-isolate strategy is likely not practicable in most countries, and a degree of social distancing, ranging up to full lockdown, is the main public-health tool to mitigate the COVID-19 pandemic. Guided by reliable surveillance data, distancing should be continuously optimised down to the lowest sustainable level that guarantees a low and stable infection rate in order to balance its wide-ranging negative effects on public health. The qualitative mixture of social-distancing measures also needs to be carefully optimised in order to minimise social costs.

## Introduction

The rapid spread of the coronavirus disease 2019 (COVID-19) pandemic led to the widespread introduction of social distancing ranging up to full lockdown. As countries are considering scaling back distancing amidst considerable scientific uncertainty, a clear overall strategy for the management of the COVID-19 pandemic is often lacking. Here, we try to conceptualise such a strategy – one that is robust to the uncertainties and to the implicit assumptions behind the various public-health actions proposed. To do that, it is important first to clarify some fundamental facts of the case.

## Herd immunity is an end state, not a strategy

Herd immunity has been widely bashed as the ‘failed strategy’ that the UK followed before changing tack and imposing a national lockdown. The ensuing controversy has all but poisoned this scientific term, which just refers to a state where the number of people immune in a population is so high that a pathogen cannot find enough susceptibles to infect and gradually dies out. In reality, herd immunity is the only possible endgame of the COVID-19 pandemic. Given the worldwide extent of viral spread and the large degree of asymptomatic or mildly symptomatic transmission [1], containing the severe acute respiratory syndrome coronavirus 2 (SARS-CoV-2) virus in the same way that Ebola or SARS-CoV-1 were managed is beyond the realms of possibility. Therefore, the pandemic will only definitively end once herd immunity is reached, whether that be through vaccination, natural infection or a mixture of the two [2].

Furthermore, even a modest degree of population immunity, at levels below those required for herd immunity, still results in a proportional reduction in the transmissibility of the pathogen. Therefore, it does help to bring the effective reproduction (R) number <1 and reverse the course of the pandemic, alongside proper control measures. This suggests that countries that had more infections in the first pandemic wave may face fewer challenges in controlling a potential second wave, and vice versa.

An important caveat is that the duration of protective immunity after natural infection with SARS-CoV-2 is not currently known, and is one of the most urgent questions for research. Antibodies have been shown to last for at least a few months [3], and T-cell responses are likely to persist for several years more [4,5]. Nevertheless, if people can be reinfected with SARS-CoV-2 within a few years, the virus could become endemic like other seasonal respiratory viruses (e.g. influenza, respiratory syncytial virus, etc.). In such a case, herd immunity might only be achievable through vaccination, which might have to be repeated in order to sustain immunity levels.

## Lockdowns are not free in terms of public health

The dilemma between maintaining or lifting lockdowns is often counterproductively framed as a contrast between population health and the economy. In the absence of effective therapeutics and vaccines, lockdowns are intended to prevent mass casualties from the rapid introduction of a virus in an immunologically naïve population, especially in countries with a poor health infrastructure, limited surge capacity and/or social inequalities. However, lockdowns themselves have a variety of negative effects on health, which must be balanced against their benefit for controlling the COVID-19 pandemic. These effects are difficult to quantify and often overlooked. Among others, they include difficulty in accessing health care for chronic and other diseases [6], mental and physical issues due to isolation and inactivity, and the long-term effects of children being out of school [7,8]. The economic damage from the lockdown also negatively impacts public health, especially through increased unemployment and inequality [9]. Therefore, restrictive measures should be used judiciously, with a clear rationale and a reasonable expectation of net benefit in terms of population health.

Maintaining a strong lockdown indefinitely implies another strong, and usually unstated, assumption: that there will be a safe and effective vaccine available at the end of the road, produced in sufficient quantities and with a substantial proportion of the population yet uninfected. Success is not guaranteed though [10]. Real concerns exist, for example regarding antibody-dependent enhancement, and any candidate vaccine will have to be thoroughly tested before being rolled out [11]. By the time we have a vaccine, it may already be too late for it to alter the course of the COVID-19 pandemic substantially.

## One must focus on what is practicable

There is intense discussion about scaling up testing for COVID-19, in large part promoted by the World Health Organization and supported by successful examples such as in South Korea [12]. Large-scale testing is essential for strong epidemiological surveillance, which is a prerequisite for making informed public-health decisions. However, in terms of controlling the pandemic, testing can only be effective when combined with case isolation and exhaustive contact tracing. In turn, this requires immense resources, which are likely beyond reach for most countries. ‘Digital contact tracing’ using smartphone apps might be an alternative, but this creates new issues about individual privacy and human rights. For a test-trace-isolate strategy to be practicable, case numbers first need to be brought substantially down to manageable levels through social distancing. Even then, the extent of transmission by asymptomatic or otherwise unascertained COVID-19 cases is such that this strategy may not be effective in isolation [1]. It will need to be combined by some degree of social distancing, which will continue to be the main tool for controlling the pandemic and protecting public health.

## An approach for the long term, and the endgame

Controversy persists about the infection fatality rate of COVID-19, and whether it is closer to 1% or to 0.1% – a difference in deaths of an order of magnitude [13,14]. That debate often transforms itself into respective calls for indefinite maintenance or early lifting of lockdowns. An undisputable principle, however, is that the benefit from any such measures must clearly outweigh their harms to public health. It is therefore reasonable to move away from full lockdowns and calibrate social distancing down to a sustainable optimal level – one that minimises both the morbidity and mortality of COVID-19 but also the negative effects of distancing. This balance point will be continuously revised as we accumulate more scientific knowledge about COVID-19, the effectiveness of control measures and their wider impact on population health. In any case, the rational goal is not to prevent each and every SARS-CoV-2 infection at any cost, but rather to protect and maximise public health for everybody.

Such an optimisation will have to be both qualitative and quantitative. On a quantitative level, the aggregate effect of all social-distancing measures should maintain the effective R number of COVID-19 at ⩽1. This is a hard limit to ensure a stable infection rate in the population, rather than an exponentially increasing one, which would risk depleting health-care capacity, at least in some locations. If COVID-19 cases cannot be eliminated given the extent of asymptomatic transmission and continuous introductions from abroad, then a low and stable rate is the next reasonable goal. Full lockdowns were fully justified in the initial phase of the pandemic out of an abundance of caution and to bring down COVID-19 cases rapidly. Once this had been sufficiently achieved, social distancing measures could be dialled down to the lowest level that maintains R at ⩽1.

For this strategy to work, COVID-19 surveillance is paramount and needs to be substantially upscaled, alongside laboratory capacity, to cover the entire population in all geographic areas. Importantly, surveillance will continuously guide and revise the appropriate level of social distancing. If, for example, SARS-CoV-2 transmissibility decreases in the summer and rises in the autumn, surveillance indicators will reflect this, and social distancing will be calibrated to maintain a stable infection rate. Similarly, if COVID-19 cases flare up in a defined geographic area, additional targeted measures may be taken to bring the pandemic back under control.

On a qualitative level, and in order to select an optimal combination, each measure will have to be individually evaluated for both its potential benefit and its social and public health cost [15]. In this evaluation, the strong age gradient in mortality from COVID-19 needs to be taken into account [16]. A case in point is school closures, whose impact on COVID-19 transmission is uncertain and whose social costs are very high [17]. Children are the age group least vulnerable to COVID-19, and might also be less likely to infect others [18,19]. Therefore, accepting some risk of infections among children may be a reasonable compromise for the wider societal benefit of keeping schools open, with the additional side effect of building up a degree of population immunity in the safest possible way. On the other hand, very stringent measures will need to be continuously maintained in health-care facilities and elderly care homes, which are both important drivers of infection and locations where the most vulnerable are exposed. Steering infection away from those most at risk is no less important than keeping a low infection rate in order to minimise morbidity and mortality from COVID-19.

In selecting the appropriate mix of social distancing, there is often a paucity of evidence about the effectiveness of individual measures. In such a context, choices about what socio-economic activities to allow inevitably become political, based primarily on assessments about the costs to society. At the same time though, plans should be made to collect the required evidence and formally evaluate the effectiveness of each measure, for example by comparing the effective R number of the pandemic before and after its introduction.

In conclusion, using epidemiological surveillance to calibrate social-distancing measures appropriately and to achieve a low and stable infection rate, thereby minimising overall morbidity and mortality, is a reliable long-term approach to follow and maintain until the COVID-19 pandemic reaches its herd immunity endgame, hopefully through the discovery and application of a safe and effective vaccine.
